# An Adaptive Autogram Approach Based on a CFAR Detector for Incipient Cavitation Detection

**DOI:** 10.3390/s20082303

**Published:** 2020-04-17

**Authors:** Ning Chu, Linlin Wang, Liang Yu, Changbo He, Linlin Cao, Bin Huang, Dazhuan Wu

**Affiliations:** 1 College of Energy Engineering, Zhejiang University, Hangzhou 310027, China; 21960254@zju.edu.cn (L.W.); wudazhuan@zju.edu.cn (D.W.); 2 State Key Laboratory of Mechanical System & Vibration, School of Mechanical Engineering, Shanghai Jiao Tong University, Shanghai 200240, China; liang.yu@sjtu.edu.cn; 3 College of Electrical Engineering and Automation, Anhui University, Hefei 230601, China; changbh@ahu.edu.cn; 4 Institute of Advanced Technology, Zhejiang University, Hangzhou 310027, China; caolinlin@zju.edu.cn; 5 Ocean College, Zhejiang University, Hangzhou 310027, China; binhuang@zju.edu.cn; 6 State Key Laboratory of Fluid Power and Mechatronic Systems, Zhejiang University, Hangzhou 310027, China

**Keywords:** incipient cavitation, autogram method, CFAR detector, cyclic amplitude model, square-envelope spectrum, adaptive threshold, feature extraction, autocorrelation, predictive maintenance

## Abstract

Cavitation failure often occurs in centrifugal pumps, resulting in severe harm to their performance and life-span. Nowadays, it has become crucial to detect incipient cavitation ahead of cavitation failure. However, most envelope demodulation methods suffer from strong noise and repetitive impacts. This paper proposes an adaptive Autogram approach based on the Constant False Alarm Rate (CFAR). A cyclic amplitude model (CAM) is presented to reveal the cyclostationarity and autocorrelation-periodicity of pump cavitation-caused signals. The Autogram method is improved for envelope demodulation and cyclic feature extraction by introducing the character to noise ratio (CNR) and CFAR threshold. To achieve a high detection rate, CNR parameters are introduced to represent the cavitation intensity in the combined square-envelope spectrum. To maintain a low false alarm, the CFAR detector is combined with the CNR parameter to obtain adaptive thresholds for different data along with sensor positions. By carrying out various experiments of a centrifugal water pump from Status 1 to 10 at different flow rates, the proposed approach is capable of cavitation feature extraction with respect to the CAM model, and can achieve more than a 90% detection rate of incipient cavitation and maintain a 5% false alarm rate. This paper offers an alternative solution for the predictive maintenance of pump cavitation.

## 1. Introduction

The centrifugal pump, as one type of fluid transport machinery, plays an important role in process industries, such as those of power, metallurgy, mining, and building, etc. A centrifugal pump belongs to a rotodynamic pump that drives fast-rotating impellers to provide transported fluids with a high pressure and high speed. Then, the driven fluids rush outward into a diffuser or volute chamber, and exit into the downstream piping system. These complex working conditions will inevitably cause cavitation-related injury directly on pump impellers and shorten the life-span. Cavitation is a physical phenomenon in which pressure differentials within flowing liquids can quickly lead to the massive formation of vapor-filled cavities or bubbles [1,2,3]. The energy released by bubble collapse will suddenly damage pump assets and peripherals, such as blades, seals, valves, pipelines, etc. With cavitation fast evolving from bubbles to clouds, it will generate harmful vibration and impulsive noise, and then cause blade erosion and seal damage, thus seriously restricting the pump efficiency and shortening the life-span for a long run. As a consequence, such cavitation failure of critical pumps will result in costly downtime, system failure, and fatal accidents for all process industries.

From the perspective of predictive maintenance, it is highly necessary to detect incipient cavitation before cavitation clouds cause irreversible damage [4]. It is also very much expected that intelligent approaches to diagnose and predict the cavitation evolution when the pumps are running will be developed. However, challenges with regards to this still exist [5]. Firstly, in the early stage of pump cavitation, it is not easy to observe any apparent symptom, such as pressure fluctuation, head drop, strong vibration, or noise induced by incipient cavitation. Moreover, cavitation evolution is affected by many kinds of excitation sources inside kinetic flows. Therefore, the cavitation-induced signal is always modulated by various excitation components around rotating impellers and is inherently accompanied by strong background noise during pump operation.

Since the cavitation phenomenon is generally a non-stationary process, many researchers have tried multimodal sensing and innovative signal-processing in cavitation detection. He and Liu [6] carried out experimental research on the time-frequency characteristics of cavitation noise using a wavelet scalogram. Additionally, Asish et al. [7] used support vector machine (SVM) algorithms to predict flow blockages and impending cavitation in centrifugal pumps. Furthermore, Bao et al. [8] proposed a modified Empirical Mode Decomposition (EMD) technique by estimating the local mean of cavitation-related signals via the windowed average. This method effectively alleviated the unfavorable influence of noise disturbance and yielded an improvement in signal modulation extraction. Song et al. [9] proposed a novel demodulation method for rotating machinery based on time-frequency analysis and principal component analysis (PCA), as well as other extended methods in [10,11]. The demodulation method can be applied for the cavitation detection of ship propellers, which is a kind of axial pump cavitation. Stopa et al. [12] proposed Load Torque Signature Analysis by employing electrical signals from the motor to estimate pump torque and to determine the cavitation occurrence and intensity through spectrum information. Sun et al. [13] conducted cavitation experiments and Hilbert–Huang Transformation (HHT) on vibration and current signals. The root mean square of the Intrinsic Mode Function (IMF) in the current signal is sensitive to cavitation. They [14] also proposed a cyclic spectral analysis of vibration signals for the fault characterization of centrifugal pump cavitation. Zhou and Lu [15] proposed a multi-point noise analysis method to study cavitation noise, and used the second-generation wavelet to extract the noise energy spectrum of each microphone point, before combining the sensitive frequency bands of all measuring points to form the eigenvector and to train the Back Project (BP) network. Ramadevi [16] proposed a discrete wavelet algorithm based on db4 wavelet decomposition. By employing five-level decomposition, the various components of wavelet coefficients in each layer were obtained to detect the cavitation status. McKee et al. [17] used statistical methods, such as adaptive frequency band analysis and PCA, to extract the vibration characteristics of cavitation failure. Moreover, Dario et al. [18] employed accelerometer time-series analysis based on an auto-regressive and moving average (ARMA) method to determine the pump cavitation.

All of the above methods have successfully monitored and detected cavitation failure, but few of them can produce early warnings of incipient cavitation, and more importantly, they can hardly maintain a low probability of false alarm for the process industry requirement. According to the testing standards of centrifugal pumps, such as ANSI/HI9.6.1, ISO9906-2012, and ANSI/API610, a cavitation problem cannot be confirmed unless the inlet flow at the testing point remains steady and the output head drops by 3% of the original value. However, this evidence cannot be used to predict incipient cavitation, because, at the very beginning of cavitation occurrence, there are no distinct changes in head drop or efficiency loss. Moreover, other symptoms, such as strong vibration and impulsive noise, are not very noticeable during incipient cavitation. As long as the output head drops by 3% and the efficiency falls, although abnormal noise and vibration are visible, pump cavitation has already reached a serious status. In addition, the boundary between different cavitation statuses is not distinct. These are the reasons why it is difficult for most of the aforementioned state-of-the-art methods to detect incipient cavitation during pump operation.

To overcome the above difficulties in incipient cavitation detection, some researchers have improved spectral kurtosis methods that measure the intensity of the transient impact caused by cavitation bubble collapse. An early version of spectral kurtosis was first introduced by Dwyer [19], but not in the field of gear box diagnosis. In later research, a refined version was successfully proposed by Antoni and Randall [20], and was thus widely applied in many fields of rotary machine diagnosis [21,22]. Kurtosis is defined as the measure of thickness or heaviness of the random variable distribution along its tail. According to the kurtosis value, the distribution can be classified into three categories. The distribution with kurtosis equal to 3 is known as mesokurtic, such as a normal distribution. If the kurtosis is less than 3, the distribution is called platykurtic, and has a shorter and thinner tail and a lower and broader peak compared to a normal distribution. If the kurtosis is greater than 3, the distribution is leptokurtic, and has a longer and fatter tail and a higher and sharper peak than those of a normal distribution. In general, the stronger the transient impact of the signal, the greater its kurtosis becomes. In 2006, Antoni [23] developed Spectral Kurtosis (SK) based on improved time-frequency analysis. SK has since become one of the dominant methods used to demodulate the spectrum band in which the envelope signal has the maximum impulsivity or kurtosis value. All the values of SK for each central frequency and related spectrum band can be shown defectively in a colorful 2D map called a Kurtogram. For industry application, Fast Kurtogram (FK) was further proposed by Antoni [24] based on a multirate filter-bank, in order to simplify the complex computation of the conventional Kurtogram. Nowadays, SK is widely used to diagnose bearing faults, gear box failure, and engine deficiency. For the presence of strong noise, an improved Kurtogram was proposed by Lei et al. [25] based on Wavelet Packet Transform (WPT) to enhance the spectrum resolution of transient impulses. However, there is little literature [8,12] reporting Kurtogram-based methods used to detect pump incipient cavitation. This is because a Kurtogram cannot precisely estimate the kurtosis values of cavitation signals involving repetitive impacts. The spectral Kurtosis performance declines in the case of a low signal-to-noise ratio (SNR), or in the presence of non-Gaussian noise. Unfortunately, these aforementioned cases commonly exist within the cavitation phenomenon.

To improve the Kurtogram performance against intense impulses of cavitation, several innovative methods have recently been exploited, such as the Protrugram, Infogram, and Autogram. Barszcz et al. [26] argued that the Kurtogram fails to identify repetitive transients, since the kurtosis value of the Kurtogram decreases with the increase of impulse repetition. To overcome this drawback, they proposed the Protrugram to use the kurtosis of the envelope spectrum, rather than the kurtosis of the filtered signal. However, the Protrugram has to fix the demodulation bandwidth and needs to know defect frequencies, which restricts its application. To extend the Kurtogram robustness against repetitive impacts, Antoni [27] proposed the Infogram to measure both the negentropy of the square-envelope (SE) of a signal with repetitive transients, and the negentropy of the SE spectrum of the same signal. Therefore, the Infogram is able to capture the signature of repetitive transients in both time and frequency domains. Due to the complexity of the Infogram, the Autogram was recently proposed to combine the autocorrelation, kurtosis, and other statistics, in order to analyze the intensive impacts and cyclic components of cavitation-related signals [28]. It has displayed a good performance in the fault diagnosis of rotating machinery with strong impulsive interference. Different from the Kurtogram, the Autogram calculates the kurtosis of signal autocorrelation and attenuates the interference, such as random striking and non-Gaussian noise, so that it can enhance the feature extraction of fault information from cyclic frequencies and resonant spectrum bands. Furthermore, the Maximum Overlapping Discrete Wavelet Packet Transform (MODWPT) [28] in the Autogram overcomes the sensitivity of wavelet coefficients in the selection of the starting point of time series. Moreover, the Autogram employs the combined square-envelope spectrum (CSES) to extract more cyclic harmonics than the spectral kurtosis (SK) of the Kurtogram. However, few Protrugram-, Infogram-, and Autogram-based methods have been applied in pump incipient cavitation detection.

For industry applications, the frequent misjudgment of cavitation occurrence in critical pumps will interrupt the continuous operation, reduce the working productivity, and increase maintenance workloads. To avoid an unnecessarily high false alarm, the Constant False Alarm Rate (CFAR) detector can be used to reduce frequent misjudgments and improve the detection rate, since the CFAR threshold can adaptively increase or decrease in proportion based on the power level of background noise. More importantly, the CFAR threshold can conduct self-regulation according to various noise distributions, so the CFAR detector has been widely developed for radar signal detection against complicated background clutters [29,30,31]. Barkat et al. [29] employed the CFAR to automatically detect a target in a nonstationary clutter background. They pointed out that classical detection using a matched filter receiver and a fixed threshold was not applicable due to the nonstationary nature of the background noise. Therefore, adaptive threshold techniques are needed to maintain a constant false alarm rate. In cell-averaging CFAR processing, an estimate of the background noise from the leading and lagging reference windows is used to set the adaptive threshold. Lehtomaki et al. [30] combined false alarm probability-based forward methods with the cell-averaging CFAR technique to locate the outliers, and a threshold multiplier was used to scale the threshold to achieve the desired probability of a false alarm.

Therefore, this paper mainly focuses on incipient cavitation detection. Our aim is to try to provide informed decision-making and strengthen predictive maintenance in real-time. In particular, it is key to recognize incipient cavitation through a high detection rate and low false alarm. Therefore, this paper proposes a cyclic amplitude model (CAM) to reveal the cyclostationarity and autocorrelation-periodicity of pump cavitation-caused signals. In addition, the Autogram method is improved for envelope demodulation and feature extraction by introducing the character to noise ratio (CNR) and CFAR threshold.

There are two main contributions presented in this paper. To achieve a high detection rate, the improved Autogram introduces the character to noise ratio (CNR) to monitor cavitation-caused noises in the combined square-envelope spectrum (CSES). Moreover, to maintain a low false alarm, the proposed CNR parameter employs the CFAR detector to obtain adaptive thresholds in data processing from different sensor positions around one testing pump. By carrying out various experiments of a centrifugal water pump from Status 1 to Status 10 at different flow rates, the proposed approach is capable of extracting CSES features with respect to the CAM model, achieving more than a 90% detection rate of incipient cavitation, and maintaining a false alarm rate as low as 5%. In brief, this paper proposes an adaptive Autogram approach based on the CFAR criterion for pump incipient cavitation detection. The proposed approach is able to improve the detector robustness in pump cavitation diagnosis.

The structure of this paper is organized as follows: Section 2 will introduce the cyclic amplitude model of cavitation-caused signals. In Section 3, an Autogram method is compared with the Kurtogram, and the feature parameter CNR and its CFAR detector will be presented in detail. Section 4 will introduce the experimental setup of the centrifugal water pump at 80%, 100%, and 110% flow rates, respectively. Vibration signals are collected by eight accelerometers. In Section 5, the proposed CNR and CFAR thresholds will first be trained and selected by using cavitation data from Sensor 15, and then successfully applied to detect incipient cavitation measured at Sensor 17 and 19. Finally, Section 6 concludes this paper and gives perspectives.

## 2. Modeling Difficulty

**Figure 1:**
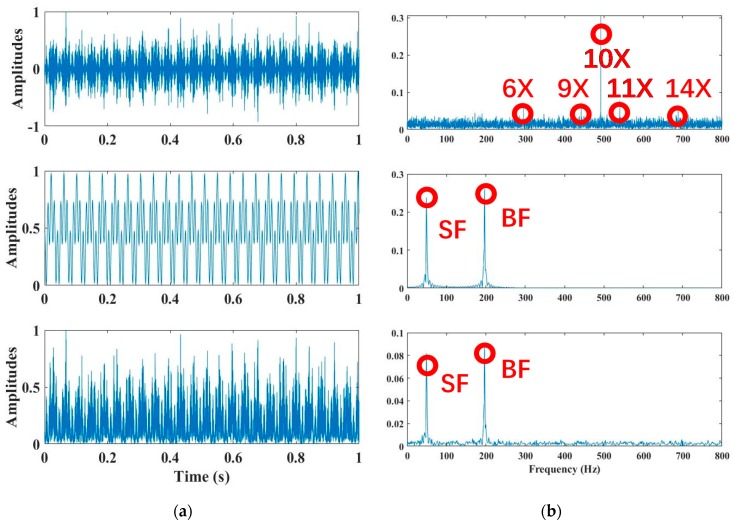
An example of the proposed cyclic amplitude model (CAM): modulation frequencies $f_{1}=49.17\;$Hz (SF), $f_{2}=4\times 49.17$ Hz (BF = 4 × SF), and modulation amplitudes $A_{1}=A_{2}=0.5.$ Narrow band carriers $f_{n}$ = 10 × SF, and their amplitude $B_{n}=0.3.\;C\left(t\right)$ is Gaussian white noise. (**a**) Simulated signal (top), autocorrelation (middle), and square-envelope (bottom); (**b**) power spectrum (top), autocorrelation spectrum (middle), and square-envelope spectrum (bottom). Note that the labels of the vertical axes represent the amplitude.

The modeling difficulty is that the measured pump cavitation-caused signal is time-varying, impulsive, and modulated by impeller rotation. Such a complicated signal has a deceptive spectrum consisting of informative cyclic components at low- and mid-frequencies, as well as amplified continuous bands at high frequencies. However, the measured pump cavitation-caused signal can still be modeled by using non-stationary statistics. Additionally, cavitation-caused vibration or acoustic signals can be approximated by cyclostationary modeling [32,33]. Owing to the rotation and reciprocation of mechanical equipment, their vibration and acoustic signals inherently exhibit cyclic stationary (cyclostationary) characteristics [33,34]. In centrifugal pumps, cavitation bubbles occur, agglomerate, and congest between inlet suck and rotating impellers [35,36]. The faster the impeller runs, the faster the cavitation evolves, and the stronger the cavitation-caused signal becomes. Since many excitation sources inside pump flows cause complex modulation effects, a cyclic amplitude modulation (CAM) model to describe the measured pump cavitation-caused signal can be expressed as

(1) $$x\left(t\right)=\left[1+\sum \;A_{i}\cos2\pi f_{i}t\right]\cdot \left[\sum \;B_{n}\cos\left(2\pi f_{n}t+\varphi_{n}\right)+C\left(t\right)\right],$$

where the first bracket$\;\left[1+\sum \;A_{i}\cos2\pi f_{i}t\right]$ plays a role in amplitude modulation or the signal envelope, and $f_{i}$ is the discrete frequency or cyclic frequency, including the shaft-rotating frequency (SF) of the pump and blade-passing frequency (BF) of the impeller, as well as their harmonics (2× SF, 3× SF etc. and 2× BF, 3× BF etc.) and coincidence frequencies; $A_{i}$ is the amplitude of the cyclic frequency component; the second bracket $\left[\sum \;B_{n}\cos\left(2\pi f_{n}t+\varphi_{n}\right)+C\left(t\right)\right]$ plays the role of a signal carrier caused by unsteady flow along the pump impeller and can be excited by fluid-solid coupling, whose spectrum contains resonant frequencies; $f_{n}\;,\;B_{n},\;\mathrm{and}\;\varphi_{n}\;$are the frequency, amplitude, and initial phase, respectively, which are also influenced by the cavitation intensity; and C(t) is a random process which depends on the cavitation evolution and is mainly generated by cavitation bubble collapses consisting of repetitive impulses, accompanied by Gaussian and non-Gaussian noise. One example of simulated signals based on the proposed CAM model is demonstrated in Figure 1. Compared with the square-envelope signal in Figure 1 (bottom), the instantaneous autocorrelation function (Figure 1 middle) is more likely a periodic signal, and the autocorrelation spectrum has a very good resolution of cyclic frequencies such SF and BF, and noise suppression is better than the square-envelope spectrum (SES) of the simulated CAM signal. Both autocorrelation and SES can reveal more cyclic frequency components than the power spectrum (Figure 1 top).

As shown in Equation (1) and Figure 1, the proposed CAM model tries to provide insights on the mechanism of cavitation occurrence, and presents a principle to identify the incipient cavitation by extracting characteristics from measured cavitation-based signals. When there is no cavitation, the envelope spectrum of the proposed CAM model mainly contains two parts, such as cyclic frequencies and wide-band noise. The former part can monitor the actual parameters of the pump operation, such as the shaft-rotating frequency, blade-passing frequency, their harmonics and coincidence frequencies, and motor electromagnetic frequencies. The latter part is induced by steady flow inside the pump, and its spectrum strength can reflect the flow distribution. When cavitation bursts out, due to the intensive collapse of many small bubbles, wide-band noise in the second part of the CAM model expands its spectrum into lower and higher frequency regions. Meanwhile, the noise spectrum amplitude will be amplified over the entire band, especially at high frequencies. In this way, the incipient cavitation can be detected if some of the cyclic frequency components in the high frequency band begin to be submerged by the arising noise spectrum. Moreover, the noise spectrum distribution can be affected by noise clutters, which are caused by the flow heterogeneity around cavitation bubbles. With bubbles rapidly developing, the bubble conglomeration and explosion will definitely lead to unsteady flows, which will probably result in nonuniform forces on pump impellers. Considering this, problems such as rotor imbalance and impeller damage, and even seal leakage bursting out, can occur due to violent cavitation. Therefore, it is highly necessary to find an effective method of incipient cavitation detection, in order to reduce pump damage and avoid performance deterioration.

In brief, without cavitation, the autocorrelation spectrum (or SES) characteristics of the proposed CAM model would be a family of comb components across the continuous spectrum, in which principal cyclic components (SF, BF, and their harmonics) sparsely distribute along low and middle frequencies, and a smooth carrier occupies high frequency bands. With cavitation occurring, some of the high-order harmonics of SF and BF begin to be buried by cavitation-caused noise in the high frequency band, while most of the cyclic components in the low and middle frequency band are the same as those without cavitation. However, severe cavitation would probably make noise clutters so intensive that most of the harmonic components become buried, even the BF, along the entire frequency band. Although SF and its low-order harmonics might be enhanced due to the blade imbalance problem caused by severe cavitation, there will no longer be a family of comb components across the continuous spectrum. The aforementioned features will be seen in the figures from the Section 3.1, Section 5.1 and Section 5.2, respectively.

## 3. Proposed Approach

### 3.1. Autogram Compared with the Kurtogram

Although the pump cavitation-caused signal is not periodic, it is generated by a hidden periodic mechanism due to the rotation of shafts and impellers, as well as the rhythmic behavior of bubbles and flows. This sort of signal is regarded as a cyclostationary signal, since the signal’s statistical moments vary cyclically with time. The n-order cyclostationary signal has an important property in that its n-th order moments are periodic, as depicted in Figure 1 (middle and bottom). For example, the vibrations produced by pump cavitation include a series of repetitive impulses. In fact, the proposed CAM model in Equation (1) takes the cavitation-caused vibration as the second-order cyclostationary signal. Therefore, the proposed CAM model can reveal two essential features of the pump cavitation signal. One is that it has a periodic autocorrelation in the time domain. The other is that it has a cyclic amplitude-modulation spectrum.

This work is motivated by the aforementioned features modeled by CAM in Equation (1), and employs periodic autocorrelation to characterize the second-order cyclostationarity of pump cavitation-caused signals. An unbiased autocorrelation is used to analyze the repetitive impulses in incipient cavitation. Similar to the Kurtogram method, the proposed approach prefers no prior knowledge of signal parameters. On the contrary, the proposed CAM model can help the inverse problem to infer cavitation-caused signal parameters from measured pump vibrations.

The procedure of the inverse problem aims to find out the best spectrum band and center frequency (narrowband carrier) for envelope demodulation of the CAM model, and then extracts the features of cavitation occurrence from the square-envelope spectrum and autocorrelation. In general, the Kurtogram or Fast Kurtogram computes the kurtosis of the measured signal that is filtered in the different spectrum bands (nodes for short), and then selects one of nodes in which the filtered signal possesses the largest kurtosis. Finally, the selected spectrum band is taken as the envelope spectrum, and demodulated signals can be obtained by inverse Fourier Transform (FT), so the feature extraction from the envelope spectrum can detect the hidden non-stationarities of measured signals. Here, Kurtosis is used to describe the degree of steepness of a random distribution, as defined in Equation (2). It has been widely used to detect the impact fault in rotating machinery [20,21,22,23,24,25,26].

(2) $$Kurtosis\left(x\right)=\frac{\sum_{i=1}^{N}{\left(x\left(t_{i}\right)-\mu_{x}\right)}^{4}}{{\left(\sum_{i=1}^{N}{\left(x\left(t_{i}\right)-\mu_{x}\right)}^{2}\right)}^{2}},$$

where $x\left(t_{i}\right)$ is the signal collected at the time of $t_{i}=i/f_{s}$, i is the sampling index$,\;f_{s}$ is the sampling frequency, $\mu_{x}$ is the average value of the signal, and N is the total number of sampled data. As pointed out in [26,27,28], the Kurtogram performance is limited in several conditions, i.e., low SNR or strong non-Gaussian noise such as randomly distributed impulses.

**Figure 2:**
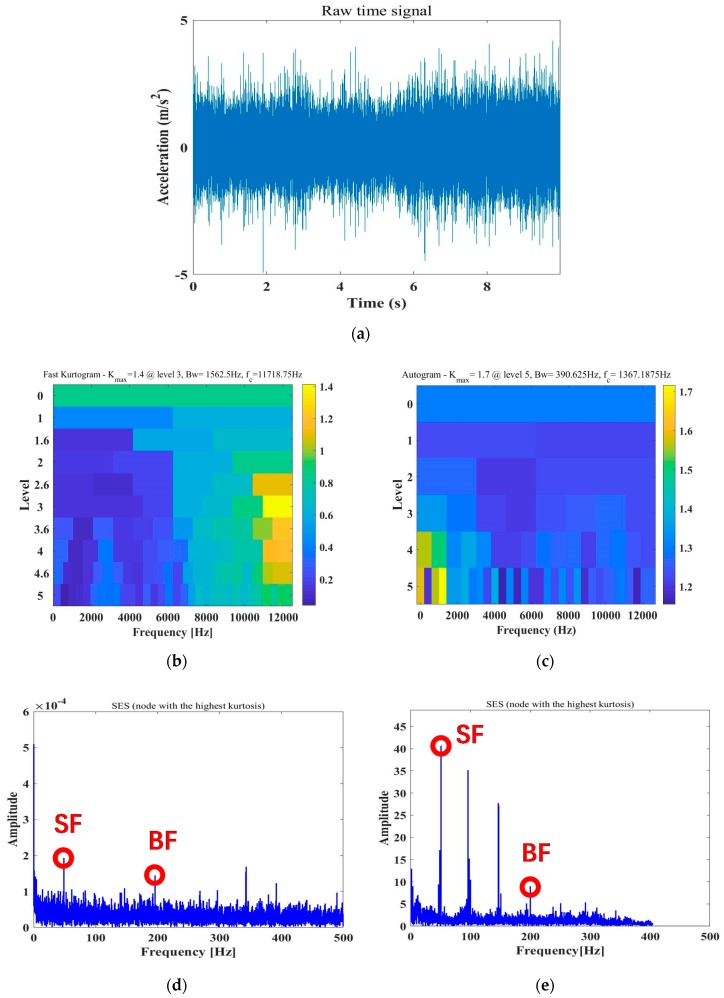
Autogram vs. Kurtogram of pump vibration signals at Status 7 at a 100% flow rate, with real data measured at Sensor 15: (**a**) Acceleration signals of pump vibrations from Sensor 15 at Status 7 at a 100% flow rate; (**b**) Kurtogram of real data. The brightest node indicates the biggest kurtosis; (**c**) Autogram of real data. The brightest node indicates the biggest kurtosis; (**d**) Autogram of real data. The brightest node indicates the biggest kurtosis; (**e**) square-envelope spectrum related to the brightest node.

Compared with the Kurtogram method, as shown in Figure 2, this paper extends the Autogram method for incipient cavitation detection by selecting the optimal spectrum band for envelope demodulation in the harsh case of low SNR or repetitive noise. The Autogram evaluates the kurtosis of unbiased autocorrelation of the square-envelope signal. Similar to the Kurtogram colormap presented in Figure 2b, all kurtosis values obtained by the Autogram in different spectrum bands (nodes) are demonstrated in a colormap in Figure 2c. As the Autogram shows in Figure 2b, the color scale is proportional to the kurtosis value, and the vertical and horizontal axes represent the levels of MODWPT decomposition and frequency, respectively [28]. Moreover, the brightest node in the Autogram colormap refers to the spectrum band, in which the filtered signal has the biggest kurtosis, and the brightest node not only reflects the maximum kurtosis value, but also the band width and the center frequency $f_{c}$ (regarded as a sinusoidal carrier frequency). Since the autocorrelation function is periodic (see Figure 1 middle) for the second-order cyclostationary signal modeled in Equation (1), the Autogram method can make full use of this periodicity to extend the Kurtogram application in incipient cavitation detection. Moshrefzadeh et al. [28] presented, for the first time, a compact procedure of four steps for Autogram computation, and successfully applied it to detect complicated faults of a gear box. Here, we modify their work, and provide more mathematical analysis on the Autogram procedure in the following five steps.

**Step 1. Signal decomposition.** The MODWPT is used to decompose time signals into different levels of spectrum bands and center frequencies, as formulated in Equations (3) and (4).

(3) $$x^{l}\left(t,n\right)=\mathrm{MODWPT}\;\left[x\left(t\right)\right],$$

where l is the index of levels, as shown in the vertical axis of Figure 2b,c, l=0,1,2,…,L, and L is the total number of levels. n is the index of nodes (spectrum bands), $n=1,2,3,\ldots ,2^{l}$. The spectrum bandwidth Bw(l) at the l-th level, center frequency $f_{c}$ (l,n) at the l-th level, and n-th spectrum band are obtained respectively as:

(4) $$Bw\left(l\right)=\frac{1}{2^{l+1}}f_{s},\;f_{c}\left(l,n\right)=\left(n-\frac{1}{2}\right)Bw=\left(n-\frac{1}{2}\right)\frac{1}{2^{l+1}}f_{s},$$

where $f_{s}$ is the sampling frequency.

**Step 2**. **Unbiased autocorrelation** of square-envelope signals. The square-envelope signals of the filtered signals $x^{l}\left(t,n\right)$ in Equation (3) at each level and node are calculated, and the autocorrelation of the square-envelope is then obtained, as formulated in Equation (5) and Equation (6).

(5) $$X^{l}\left(t,n\right)=corr\left({\left|Hilbert\left[x^{l}\left(t,n\right)\right]\right|}^{2},\;{\left|Hilbert\left[x^{l}\left(t,n\right)\right]\right|}^{2}\right),$$

where $X^{l}\left(t,n\right)$ is the square-envelope of the signal $x^{l}\left(t,n\right)$ at the l-th level and n-th node, corr(.) denotes the correlation operator, operator Hilbert[x(t)] is defined as Hilbert[x(t)]=x(t)+j∗H[x(t)], and H[x(t)] denotes the Hilbert transform of signal x(t). Therefore, the unbiased autocorrelation ${\hat{R}}^{l}{}_{xx}\left(\tau ,\;n\right)$ of the square-envelope $X^{l}\left(t,n\right)$ can be defined as:

(6) $${\hat{R}}^{l}{}_{xx}\left(\tau_{q},\;n\right)=\frac{1}{N-q}\sum_{i=1}^{N-q}\;X^{l}\left(t_{i},\;n\right)\;X^{l}\left(t_{i}+\tau_{q},\;n\right),$$

where E{·} is the mathematical expectation; $t_{i}=i/f_{s}$ with i = 1, 2, …, N−1, is the instantaneous time; $\tau_{q}=q/f_{s}$ is the delay time, with q=0, 1,…,N−1; and N is the total amount of sampling data.

**Step 3**. **Autogram colormap**. The kurtosis value of unbiased autocorrelation ${\hat{R}}^{l}{}_{xx}\left(\tau ,\;n\right)$ is calculated in Equation (6) at each level and node, and the Autogram colormap is developed, similar to the Kurtogram. The Autogram is formulated as

(7) $$Autogram\left[X,l,n\right]=\left[Kurtosis\left(X,l,n\right),l,n\right],\;Kurtosis\left(X,l,n\right)=\frac{\sum_{q=1}^{\frac{N}{2}}\left[{\hat{R}}^{l}{}_{xx}\left(\tau_{q},\;n\right)-\;\min({\hat{R}}^{l}{}_{xx}\left(\tau_{q},\;n\right)\right){]}^{4}}{{\left[\sum_{q=1}^{\frac{N}{2}}\left[{\hat{R}}^{l}{}_{xx}\left(\tau_{q},\;n\right)-\min({\hat{R}}^{l}{}_{xx}\left(\tau_{q},\;n\right)\right){]}^{2}\right]}^{2}},$$

where Autogram[X,l,n] means the Autogram colormap made up of Kurtosis of the ${\hat{R}}^{l}{}_{xx}\left(\tau_{q},\;n\right)$l-th level and n-th node, and min(.) denotes the minimization operator. It is noted that the Autogram is the colormap including all the kurtosis values of unbiased autocorrelation of the square-envelope. Compared to the minimization operator in Equation (7), there are other ways to extend the kurtosis value calculation. For example, in Equation (7), the “low threshold” or “high threshold” Autogram can be obtained by respectively setting high or low thresholds for the unbiased autocorrelation of the square-envelope signal.

**Step 4. Square-envelope spectrum (SES)** of unbiased autocorrelation. The Autogram colormap is searched, the square-envelope with the highest kurtosis value is selected, and the square-envelope spectrum (SES) is calculated as

(8) $$X^{l_{max}}\left(f,\;n_{max}\right)=\left|\;DFT\left(X^{l_{max}}\left(t,\;n_{max}\right)\right)\;\right|,$$

where $X^{l_{max}}\left(f,\;n_{max}\right)$ is the SES with the highest kurtosis; | . | denotes the absolute operator; DFT(.) denotes the Discrete Fourier Transform; and $l_{max}$ and $n_{max}$ are the indices corresponding to the maximum kurtosis value, related to $Kurtosis\left(X,l_{max},\;n_{max}\right)$ of unbiased autocorrelation ${\hat{R}}^{l}{}_{xx}\left(\tau ,\;n\right)$ at the $l_{max}$ level and $\;n_{max}$ node defined in Equation (7).

The band with the highest kurtosis value is selected for subsequent processing; in that case, some fault information may be ignored. To avoid missing important information, it is better to set the kurtosis threshold, which is half of the maximum kurtosis value within the decomposition level, as shown in Step 5.

**Step 5. Combined square-envelope spectrum (CSES).** All of the SES in different levels whose kurtosis values are greater than the threshold are normalized and selected SES are combined to obtain the CSES and average CSES as

(9) $$\begin{array}{c} CX\left(f,l\right)=\sum_{n\in N_{l}}\;\bar{X^{l}\left(f,n\right)},\\ N_{l}=\left\{n\;|\;n=\arg_{\left(n=1,2,3,\ldots ,2^{l}\right)}\{Kurtosis\left(X,l,n\right)>\frac{1}{2}Kurtosis\left(X,l_{max},\;n_{max}\right)\}\right\},\\ \bar{CX\left(f\right)}=\frac{1}{L}\sum_{l=1}^{L}CX\left(f,l\right), \end{array}$$

where CX(f,l) is defined as the combined square-envelope spectrum (CSES), and is also a colormap of the square-envelope spectrum at different decomposition levels;$\;\bar{X^{l}\left(f,n\right)}$ is the normalized SES by its norm according to $X^{l}\left(f,n\right)$ obtained in Equation (8); $N_{l}$ is the ensemble of node indices, whose item satisfies that the corresponding kurtosis value Kurtosis(X,l,n) is greater than the defined threshold $\frac{1}{2}Kurtosis\left(X,l,\;n_{max}\right)$;$\;n_{max}$ is the node index referring to the maximum $Kurtosis\left(X,l_{max},\;n_{max}\right)$; and $\bar{CX\left(f\right)}$ means the average CSES.

**Figure 3:**
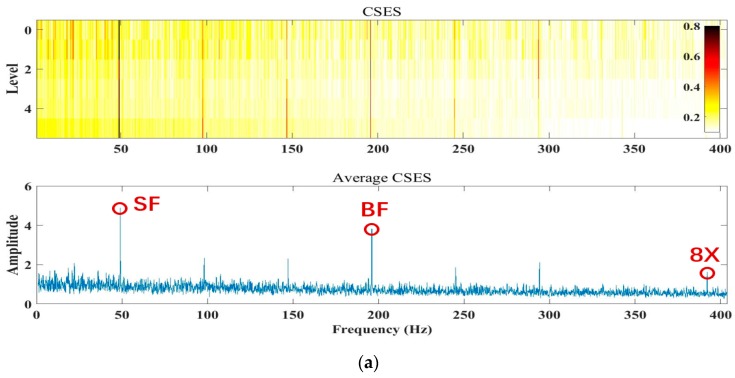

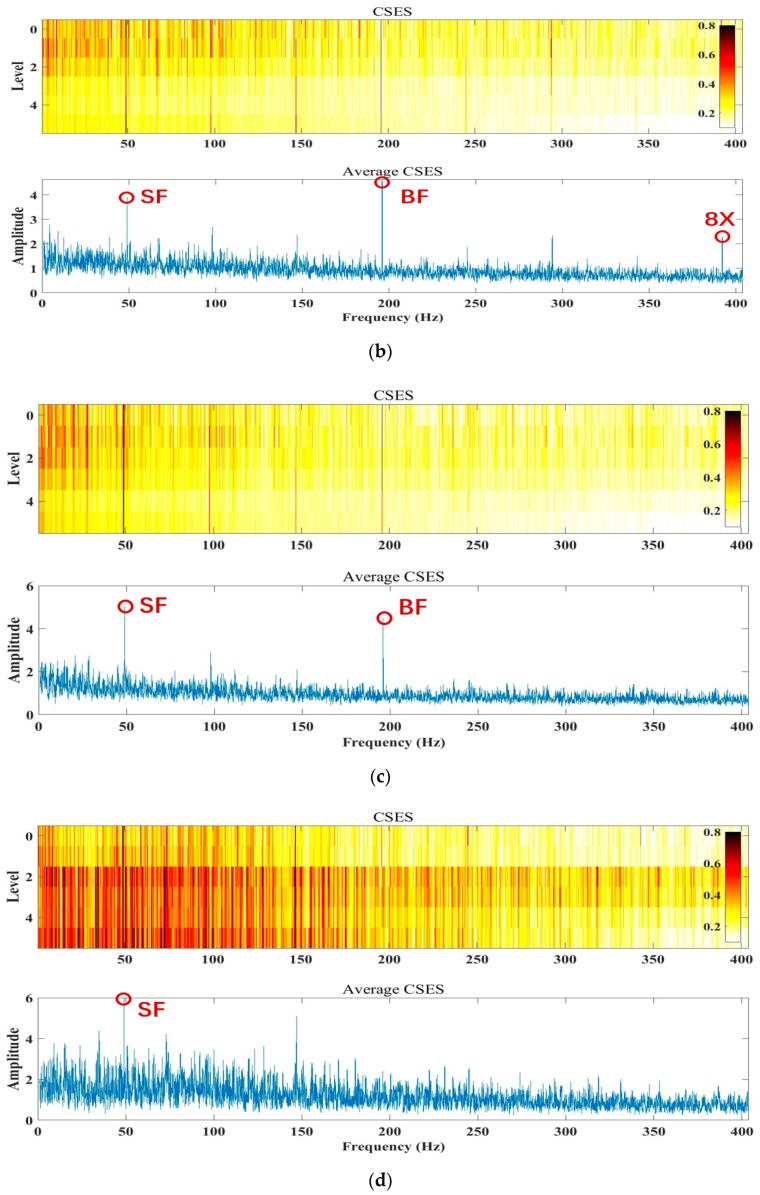
The combined square-envelope spectrum (CSES) and average CSES of pump vibration signals measured at Sensor 15, at Status 1, 5, 7, and 10, at a 100% flow rate: (**a**) CSES and average CSES at Status 1: No cavitation; (**b**) CSES and average CSES at Status 5: No cavitation; (**c**) CSES and average CSES at Status 7: No cavitation; (**d**) CSES and average CSES at Status 10: Severe cavitation.

In Figure 3, a comparison of four groups of the CSES colormap defined in Equation (9) related to Status 1, 5, 7, and 10 of cavitation experiments is presented. The real data were obtained from the pump vibration signals measured at Sensor 15 with a 100% flow rate. As pointed out by the proposed CAM model, without cavitation at Status 1 and 5, the spectrum characteristics are a family of comb components across the continuous spectrum. The SF, BF, and their harmonics sparsely distribute along low and middle frequencies, and the smooth carrier occupies high-frequency bands. With cavitation occurring at Status 7, the 8X SF begins to be buried by cavitation-caused noise, while most of the cyclic components remain the same as those at Status 1 and 5. However, severe cavitation at Status 10 makes noise clutters so intensive that most of the harmonic components, as well as the BF, are buried.

### 3.2. Proposed Feature Parameter CNR

With cavitation rapidly developing, the cavitation-caused noise intensity will increase and submerge some of the high-frequency components, as the proposed CAM model has pointed out in Equation (1). Such a fact can be represented by the CNR parameter defined in Equation (10). Similar to the definition of SNR, the CNR is proposed to compare the intensity of cyclic frequencies (numerator) with the intensity of cavitation-caused noise (denominator) in the SES or CSES spectrum.

(10) $$CNR=\frac{1}{2}\log\frac{P_{c}}{P_{n}}=\log\frac{\sqrt{\sum_{j\in K}{\bar{\;CX\left(f_{j}\right)}}^{2}}}{\sqrt{\sum_{i=1}^{Q}{\bar{\;CX\left(f_{i}\right)}}^{2}-\sum_{j\in K}{\bar{\;CX\left(f_{j}\right)}}^{2}}}\;,$$

where the numerator $P_{c}=\sum_{j\in K}A_{j}^{2}$ is the estimated power of cyclic frequency components in SES or the average CSES spectrum $\bar{CX\left(f\right)}$ derived in Equation (9); K is the the ensemble of indices of cyclic frequencies; the denominator $P_{n}=\sum_{i=1}^{Q}{\bar{\;CX\left(f_{i}\right)}}^{2}-\sum_{j\in K}{\bar{\;CX\left(f_{j}\right)}}^{2}$ is the estimated power of residue noise caused by the pump working or pump faults like cavitation; log(.) means the logarithm operator based on 10; $A_{j}$ is the amplitude of the jth cyclic frequency; $\sum_{j\in K}A_{j}^{2}$ is the sum of the square amplitudes of selected cyclic frequencies; ${\bar{\;CX\left(f_{j}\right)}}^{2}$ is the sum of the square amplitudes of all cyclic frequencies; Q is the total number of cyclic frequencies in the selected spectrum band; $A_{sf}$ is the SF amplitude; and $A_{bf}$ is the BF amplitude.

As the cavitation becomes stronger and stronger, the residue noise influenced by cavitation in the SES or average CSES spectrum increases faster than most of the cyclic frequency components, so the CNR value in Equation (10) becomes smaller as cavitation becomes worse. That is to say that the smaller the CNR is, the more possible it is that cavitation happens. The CNR at a no-cavitation status is relatively higher than the one at a cavitation status. The CNR at severe cavitation is higher than that at incipient cavitation.

To make the proposed CNR parameter more sensitive to cavitation outbreak, we firstly take the square root and logarithm operators, and thus enlarge the CNR ratio gap between the normal and cavitation status. In the calculation of the cavitation-caused noise intensity, we remove the influences of SF and BF, since they are the dominant characteristic frequencies with high amplitudes. Moreover, strong SF and BF components still exist in the square spectrum at each decomposition layer in the CSES colormap. Even though cavitation would enhance the SF and BF more or little, as the proposed CAM model in Equation (1) indicates, these increments might not be very considerable compared with the original strengths of SF and BF components. Consequently, SF and BF are not the best features to represent the cavitation occurrence.

For the selection of characteristic frequency components, we use wavelet transformation (WT) to get the wavelet coefficient matrix of square-envelopes mentioned in Step 4 of Section 3.1, and then employ PCA to extract the principal wavelet coefficients. In this way, informative characteristic frequencies are selected through the combination of WT and PCA results, so that the ensemble K of the index of characteristic frequencies can be determined. Therefore, the CNR parameter defined in Equation (10) can be calculated.

**Figure 4:**
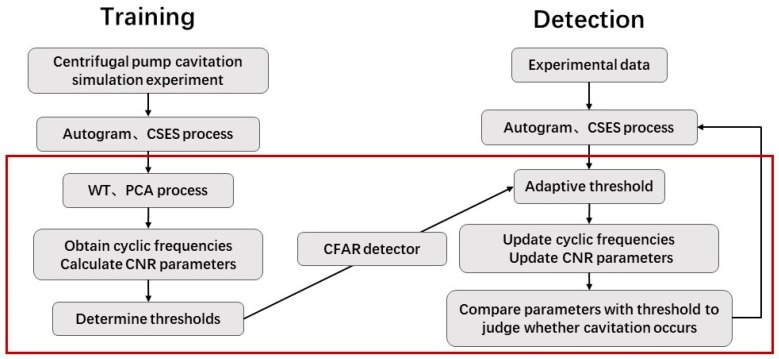
The flowchart of the proposed approach for detecting pump incipient cavitation.

As for the selection of the CNR threshold in judging cavitation, different sensor positions installed at one pump will also lead to different CNR thresholds. Before cavitation detection, it is necessary to carry out a series of experiments under a normal status, incipient cavitation, and severe cavitation. First, the actual CNR values are obtained from the SES or CSES spectrum by employing the Autogram method described in Section 3.1, and the CNR values are then matched with the normal status, incipient status, and cavitation status, respectively, by clustering analysis. Finally, the CNR thresholds are selected according to classified CNR values by maximizing the detection rate of cavitation. The above results are consistent with the incipient cavitation characteristics modeled by the proposed CAM model in Equation (1). The proposed CNR and CFAR threshold can be implemented by the flowchart in Figure 4.

### 3.3. Proposed Adaptive CFAR Threshold for CNR

**Figure 5:**
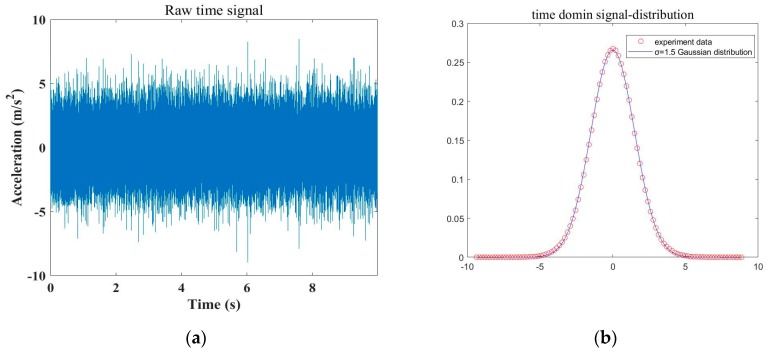

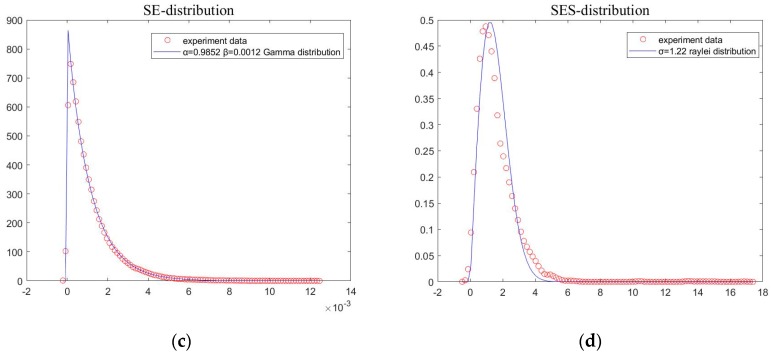
Pump vibration signals x(t), and different distribution fittings for the signal x(t), square-envelope (SE) X(t), and square-envelope spectrum (SES) X(f), respectively. (**a**) Measured vibrational acceleration signals from a centrifugal pump, noted as random signals x(t); (**b**) Gaussian distribution fitting for random signals x(t), and $x\left(t\right)\sim N\left(0,\sigma^{2}\right)$, with σ=1.5 and fitting error RMSE = 0.0013; (**c**) Gamma distribution fitting for square-envelope (SE) X(t), and X(t)~Gamma(α,β), with α=0.9852, β=0.0012, and RMSE = 4.0892, where X(t) is derived from Equation (5); (**d**) Rayleigh distribution fitting for the square-envelope spectrum (SES) X(f), and X$\left(f\right)\sim Rayleigh\left(\sigma^{2}\right)$, with σ=1.22 and MSE = 0.0296, where X(f) is derived from Equation (8).

More importantly, in order to maintain a low false alarm rate and avoid any missing detection, we can use the CFAR detector to set an adaptive threshold according to the cavitation-caused noise intensity. Assuming that the noise in the SES or CSES spectrum follows the Rayleigh distribution, the noise probability density function p(z) can be given as

(11) $$p\left(z\right)=\frac{z}{\sigma^{2}}e^{-\frac{z^{2}}{2\sigma^{2}}},$$

where the average noise power is $P_{n}=\int_{\eta }^{+\infty }z^{2}\;p\left(z\right)\;dz=2\sigma^{2}$, and the parameter $\sigma^{2}$ can be estimated by using curve fitting of the statistical histogram in the SES or average CSES spectrum. In Figure 5, an example of pump vibration signals x(t), and different distribution fittings for the signal x(t) which is fitted by the Gaussian distribution, the square-envelope (SE) X(t) which is fitted by the Gamma distribution, and the square-envelope spectrum (SES) X(f) which is fitted by the Rayleigh distribution, respectively, are demonstrated. p(z) is just the distribution fitting for SES X(f), as shown in Figure 5.

Owing to proper Rayleigh distribution fitting for SES X(f), the probability of a false alarm $P_{fa}\;$can be expressed by Equation (11) as follows:

(12) $$P_{fa}\left(\eta ,\;\sigma^{2}\right)=\int_{\eta }^{+\infty }p\left(z\right)\;dz=\int_{\eta }^{+\infty }\left(\frac{z}{\sigma^{2}}e^{-\frac{z^{2}}{\sigma^{2}}}\right)dz,$$

where $P_{fa}$ is the defined false alarm rate with $0<P_{f}\ll 1$, η is the expected threshold for satisfying a given $\;P_{fa}$ value, $P_{fa}\left(\eta ,\;\sigma^{2}\right)$ is the function of the threshold and noise power, and $e^{\left(.\right)}$ is the exponential function. According to the property of the Rayleigh distribution, the adaptive threshold value η can be obtained for a given $P_{fa}$ value from Equation (12) as

(13) $$\eta =-P_{n}\;\ln P_{fa}=-2\sigma^{2}\;\ln\;P_{fa},$$

where ln(.) is the natural logarithm operator. It has the advantage that the CFAR threshold can vary along with the noise changing. When the cavitation-caused noise becomes bigger, the threshold becomes higher, so that the probability of a false alarm $P_{fa}$ can remain constant. More importantly, the CFAR detector can regulate the threshold reasonably, according to the different probability density functions p(z) of the noise in the SES or average CSES spectrum. Namely, p(z) can be of various distributions, such as Rayleigh, Gaussian, Cauchy, Student-t, Gamma, $X^{2}$, and other continuously random distributions.

According to Equations (10) and (13), the proposed CNR threshold and CFAR threshold η under a given false alarm rate $P_{fa}$ can be formulated by

(14) $$CNR_{0}=\frac{1}{2}\log\frac{P_{c}}{P_{n}}=\frac{1}{2}\log\;P_{c}-\frac{1}{2}\log\eta \;+\frac{1}{2}\log\left(-\ln\;P_{fa}\right)\;,$$

where $CNR_{0}$ can be adaptively set by regulating the false alarm rate $P_{fa}$ according to the pump cavitation-caused noise. In this way, the CNR threshold can further be updated from one sensor ($CNR_{0}$) to another sensor ($CNR_{0}{}^{\prime }$), as follows:

(15) $$CNR_{0}{}^{\prime }=CNR_{0}-\frac{1}{2}\log\;\left(P_{c}-P_{c}^{\prime }\right)+\frac{1}{2}\log\;\left(\eta^{\prime }-\eta \right),$$

where $\eta^{\prime }$ is the updated threshold corresponding to updated $CNR_{0}{}^{\prime }$ to deal with the data from the new sensor. From Equations (14) and (13), the adaptive threshold $\eta^{\prime }\;$and updated $CNR_{0}{}^{\prime }$ can be reclusively obtained from previous η and $CNR_{0}$. The initialization of threshold η is derived from the defined false alarm rate$\;P_{fa}$. The CNR can be initialized by Equation (10) using the SES or average CSES spectrum of measured data at the referential sensor, which is Sensor 15 in the experiments of this paper.

## 4. Experimental Setup

**Figure 6:**
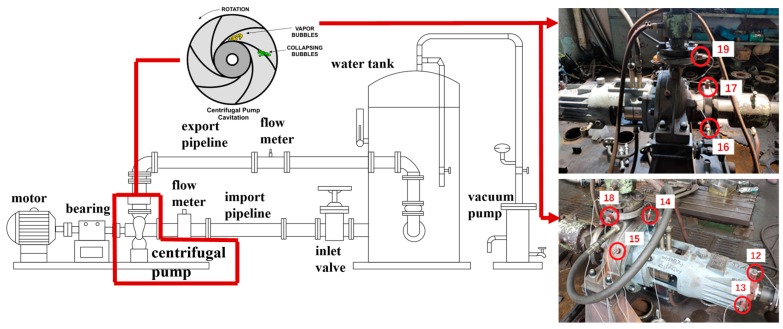
Configuration of a pump-valve test platform and the installation of eight accelerometers. In the middle part, the cavitation picture is taken from [37]. In the right part, the red circles (or ellipses) represent the accelerometers, and the number, such as 15, beside the circle is the accelerometer ID. The instruments from left to right are as follows: Motor, bearing, centrifugal pump, flowmeter, import pipeline, inlet valve, export pipeline, flowmeter, water tank, and vacuum pump.

In this study, cavitation experiments of a centrifugal water pump were carried out via a pump-valve test platform, as shown in Figure 6 and Table 1. The main parameters of a single-stage single-suction four-blade centrifugal pump are shown in Table 1. The position of the sensor has a non-negligible influence on the accuracy of pump cavitation information. Eight accelerometers were installed on the pump body and bearing end, as well as on the suction port in the radial direction, as shown in Figure 6. There are many options for the type of accelerometer, for example, type: B&K ASA-020 (B&K company, Naerum, Denmark); sensitivity: 100 mV/g; and frequency range: 4–10 kHz. In this study, we firstly processed the data collected by Sensor 15 for parameter training, which was employed to obtain the Autogram colormap, and then extracted CNR parameters and determined CFAR thresholds to label cavitation data. Following this, the raw data from other sensors were used to validate our proposed approach.

**Figure 7:**
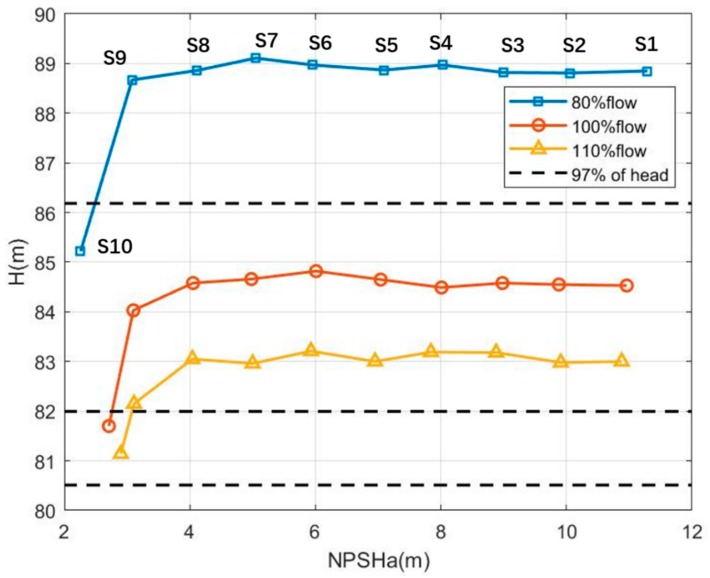
Net Positive Suction Head (NPSH)a-Head curve of the centrifugal water pump HZE80-50-250 (Delu pump company, Hangzhou, China). H denotes the Head in the vertical label. The sample point of each state is an average result of ten groups of experimental data. Each group of data contains 10 s records, including about 256,000 samplings.

In this experiment, we used the experimental routine of a constant rotating-speed and constant flow to simulate the normal and cavitation status of the centrifugal pump. The flow rates were set as 80%, 100%, and 110%, respectively. The vacuum pump and valve jointly adjusted the pressures and flows at the inlet end, so that the pump cavitation intensity was classified from Status 1 to 10. The pump status was labeled from normal operation (Status 1) to incipient cavitation (supposing Status 8) and then serious cavitation (Status 10). We calculated the head and Net Positive Suction Head (NPSH), and produced the cavitation-head curve of the centrifugal pump as shown in Figure 7, in which one curve consists of 10 samples, such as Status 10, 9, ..., and 1 (from left to right). When the flow rates were 80% (top curve) and 100% (mid curve), it was easy to judge that pump cavitation had already burst out at Status 10, since the heads of Status 10 in the two curves had already dropped by 3%, and this phenomenon confirmed the severe cavitation according to the test standard of the centrifugal water pump (ANSI/HI9.6.1, ISO9906, and ANSI/API610).

However, it was not easy for such a test standard to tell whether Status 9 or 8 in the top and middle curves had incipient cavitation or not. Following the same routine, it appeared that no cavitation at Status 10 or any other states occurred when the flow rate was 110% (bottom curve). However, in fact, strong vibrations and abnormal sounds were observed, and these facts successfully confirmed that cavitation had already occurred at Status 10 in the 110% flow case. As all the cases show in Figure 7, it is inappropriate for the conventional test standard of the centrifugal water pump to detect incipient cavitation. This is one of the reasons to employ our proposed Autogram method based on the CFAR detector.

## 5. Discussion and Analysis

### 5.1. SES Results

**Figure 8:**
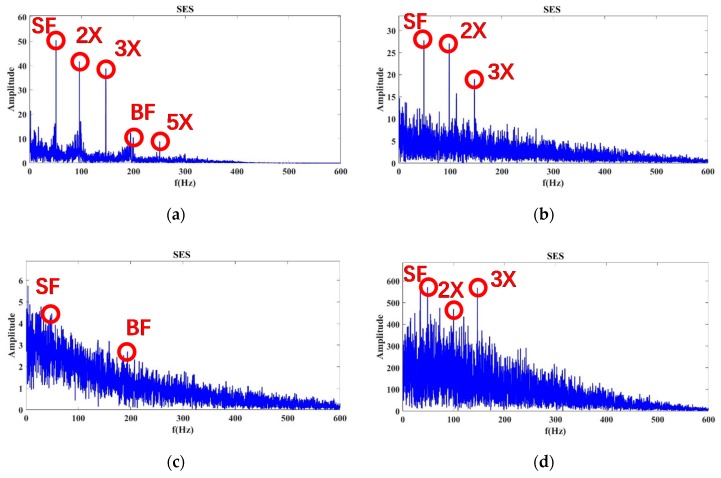
SES spectrum of the Autogram at a 100% flow rate at the accelerometer ID 15 (Sensor 15): (**a**) Status 1: No cavitation; (**b**) Status 8: Incipient cavitation; (**c**) Status 9: Severe cavitation; (**d**) Status 10: Very severe cavitation.

As shown in Figure 8, when the flow was 100%, we calculated the SES spectrum of Autogram results for Status 1, 8, 9, and 10 from the measurements of accelerometer ID 15 (Sensor 15 for short). In the SES spectrum of Status 1, SF and BF are noticeable, and the background noise is low for the SES. Status 8 can find only SF, and BF is almost submerged by noise. This can be regarded as important evidence of incipient cavitation at Status 8. The noise of Status 9 and 10 increases significantly. The SF and BF are both submerged by strong noise due to severe cavitation. According to these SES results, SF and BF are clearly visible under a normal status, such as Status 1, but they are totally drowned by severe cavitation-caused noise at Status 9 and 10. More importantly, Status 8 can be recognized as incipient cavitation due to its dominant SF and buried BF in the SES spectrum. These conclusions match the experimental conditions at Status 1, 8, 9, and 10 at the 100% flow rate well. The extracted features related to no-cavitation, incipient cavitation, and severe cavitation are quite compatible with the explanations given by the proposed CAM model in Equation (1).

**Figure 9:**
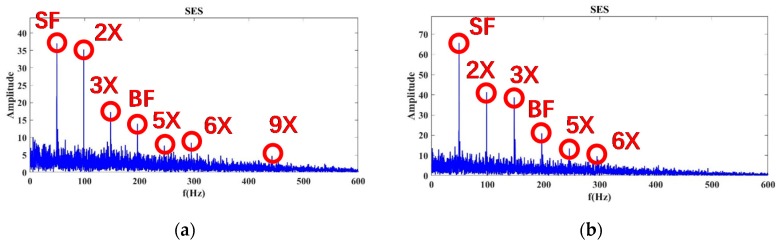

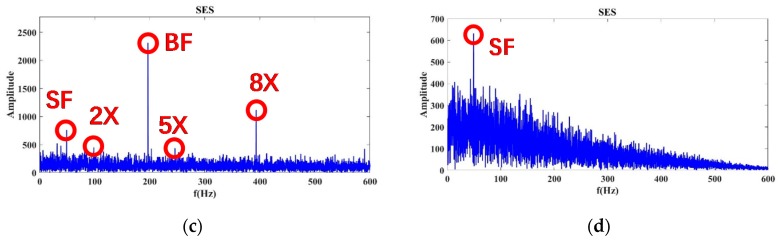
Results of the SES spectrum of the Autogram at an 80% flow rate at Sensor 15: (**a**) Status 1: No cavitation; (**b**) Status 8: Incipient cavitation; (**c**) Status 9: Incipient cavitation; (**d**) Status 10: Severe cavitation.

**Figure 10:**
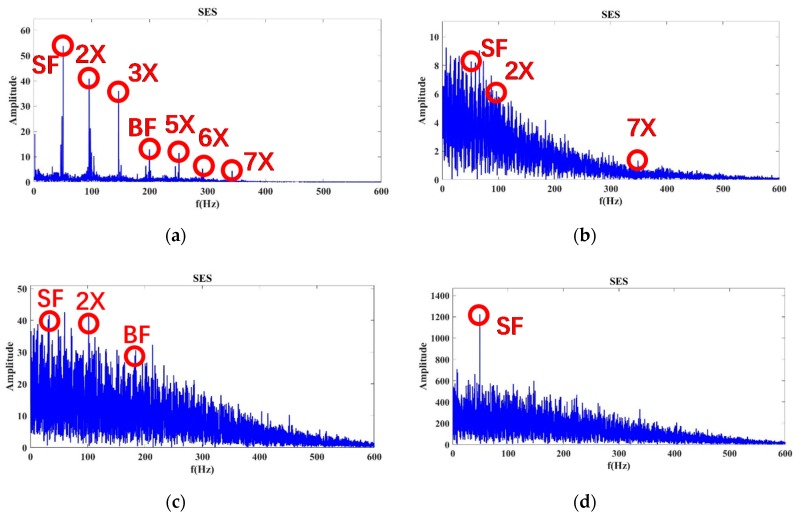
Results of the SES spectrum of the Autogram at a 110% flow rate at Sensor 15: (**a**) Status 1: No cavitation; (**b**) Status 8: Incipient cavitation; (**c**) Status 9: Severe cavitation; (**d**) Status 10: Very severe cavitation, which might cause an imbalance problem, since the shaft-rotating frequency (SF) is very much enforced.

By employing the aforementioned training strategy, the same processing was conducted for the cases of 80% and 110% flow, respectively. For the 80% flow rate shown in Figure 9, severe cavitation can be detected at Status 10. However, Status 8 and 9 exhibit typical evidence of incipient cavitation. By contrast, at the 110% flow rate shown in Figure 10, severe cavitation can be confirmed at Status 9 and 10, but Status 8 probably displays incipient cavitation. Therefore, the above experimental results show that it is more effective for the Autogram method combined with the CAM model to detect incipient and severe cavitation than conventional standards (ANSI/HI9.6.1, ISO9906, and ANSI/API610).

### 5.2. Average CSES Results

**Figure 11:**
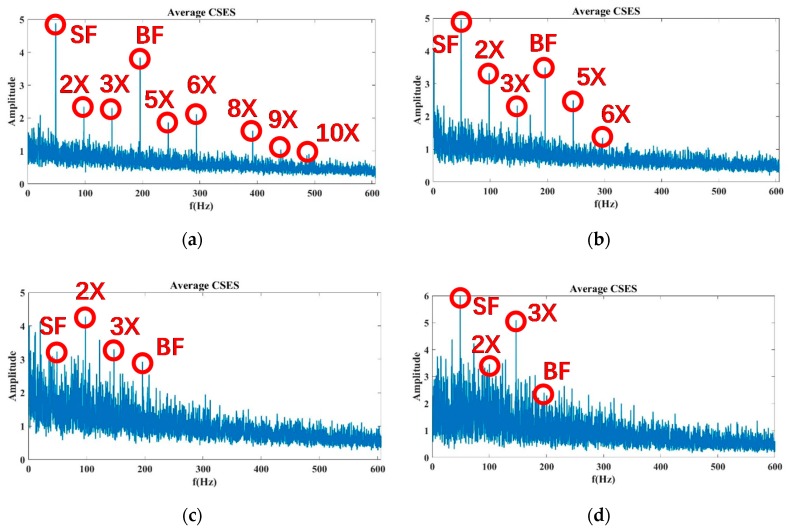
Average CSES spectrum of the Autogram at a 100% flow rate for Sensor 15: (**a**) Status 1: No cavitation; (**b**) Status 8: Incipient cavitation; (**c**) Status 9: Severe cavitation; (**d**) Status 10: Very severe cavitation.

At the 100% flow rate shown in Figure 11, the average CSES spectrum of the Autogram resulted in Status 1, 8, 9, and 10 of Sensor 15. The SF, BF, 2×, and 3× harmonics of SF can be found in these statuses. In particular, in Status 1, the 5×, 6×, and 8× harmonics of SF can be observed more clearly than those in the SES spectrum in Figure 8. However, in Status 9 and 10, these harmonic components can almost be covered by strong noise due to severe cavitation. In Status 8, the 8× harmonic has been buried, but 5× and 6× harmonics still exist, so Status 8 can be considered as incipient cavitation. Compared with the SES spectrum in Figure 8, the average CSES can provide an improved spectrum with a better high resolution and more harmonic features. Moreover, the average CSES is able to reflect the trends of noise spectra with and without cavitation just as the SES does, but more importantly, the average CSES can extract more useful harmonic information to detect the incipient cavitation than the SES.

**Figure 12:**
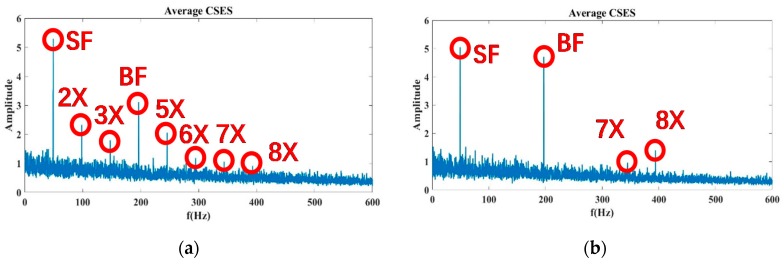

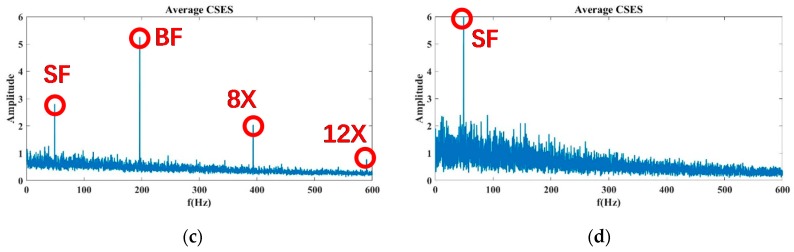
Average CSES spectrum of the Autogram at an 80% flow rate for Sensor 15: (**a**) Status 1: No cavitation; (**b**) Status 8: Incipient cavitation; (**c**) Status 9: Incipient cavitation; (**d**) Status 10: Severe cavitation.

The data with an 80% and 110% flow rate are processed in the same way. For the 80% flow rate in Figure 12, the BF information and its 5×, 6×, and 8× harmonics of Status 10 are completely submerged by noise, so that Status 10 is considered to be severe cavitation. However, Status 8 and 9, in which these harmonics are partially buried by noise, are more likely to be incipient cavitation.

**Figure 13:**
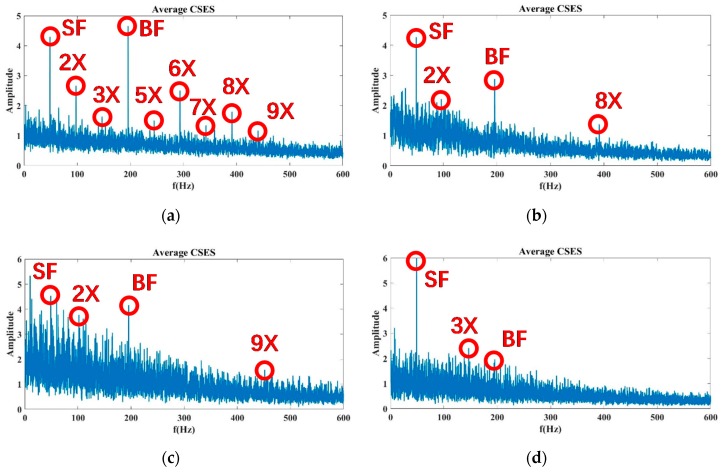
Average CSES spectrum of the Autogram at a 110% flow rate for Sensor 15: (**a**) Status 1: No cavitation; (**b**) Status 8: Incipient cavitation; (**c**) Status 9: Severe cavitation; (**d**) Status 10: Very severe cavitation.

At the 110% flow rate, as shown in Figure 13, the noise intensity of Status 8 is enhanced gradually, and the 5×, 6×, and 8× harmonics of SF information are almost submerged by the arising noise, so the evidence in the average CSES spectrum can confirm Status 8 as being incipient cavitation. Moreover, there are no high-order harmonics in Status 9 and 10 due to the problem of severe cavitation. Above all, the average CSES is more effective than the SES for detecting incipient cavitation. It can provide more useful cyclic frequency components for the CNR parameter calculation, so the CFAR detector is more robust to implement based on the average CSES spectrum of the Autogram, which is introduced later.

### 5.3. Adaptive CFAR Thresholds

According to the advantages of the average CSES spectrum, the CNR parameter can be more precisely derived from the characteristics of the average CSES spectrum than the SES, as shown in Figure 8, Figure 9, Figure 10, Figure 11, Figure 12 and Figure 13. As discussed in Section 3.3, for the selection of cyclic frequency components, we can use wavelet transformation (WT) to obtain the wavelet coefficient matrix of square-envelopes (SE), in which the SE has the biggest Kurtosis value, as derived in Step 4 of Section 3.1, and then employ PCA to extract the principal wavelet coefficients. This means that the ensemble K of the index of characteristic frequencies can be determined from the PCA and WT procedure. The above routine can be described as shown in Algorithm 1.

**Figure 14:**
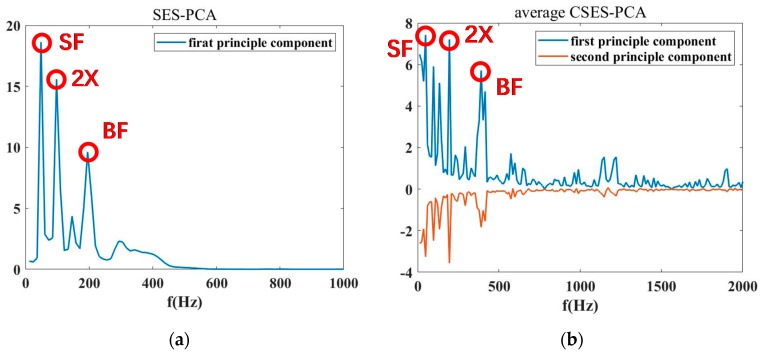
PCA selection of cyclic frequency components from SES and CSES spectra for CNRs and CNRc calculations. (**a**) The first principal component of SES-PCA. (**b**) The first and second principal components of CSES-PCA.

**Algorithm 1:** Principal component analysis (PCA) selection of cyclic frequencies for the character to noise ratio (CNR) calculation (Matlab).1: **Input**: square envelope (SE) X(t); wavelet name noted by *wname*; 2: **Part WT:**
3: wavelet coefficient matrix *WT* ← cwt(*SE, wname*) 4: **Part PCA:**
5: [m, n] ← size(*WT*), where *m* is matrix row, *n* matrix column*;* 6: covariance matrix *C* ← WT’*WT/(*m−*1)*,* where (‘) means conjugate transpose; 7: [***V****, **D***] = eig(*C, descend*), where eigenvalue vector ***D*** and eigenvector matrix ***V*** 8: *ratio = 0,* where *ratio* means cumulative contribution rate; 9: **for**
*k* = 1, …, *n*, **do** 10:    *ratio*=*ratio*+***D***(*k*)/sum(***D***)*;* 11:    **if**
*ratio* ≥ 0.85, where this threshold can be regulated according to SNR 12:     ***col*** = *k*; break; 13:    **end if** 14: **end for** 15: *nWT* ←WT* V( *:,*1*:**col***)*; where nWT* is wavelet coefficient matrix after PCA; 16: **For** i=1, 2, … ***col*, do** 17: Find out principal peaks and related frequencies in *nWT*( *:, i*)*;* 18: **end for** 19: **Output1**: Select proper peaks as cyclic frequency components from the SES and CSES in Figure 8, Figure 9, Figure 10, Figure 11, Figure 12 and Figure 13. One example is shown in Figure 14. 20: **Output2**: Calculate CNRs and CNRc in Equations (16) and (17) respectively.

Therefore, the CNR parameter defined in Equation (10) can be calculated according to K. Following the above routine, to calculate the CNR value in the SES of Figure 8, the cyclic frequencies are derived as SF and BF. For the CNR in the average CSES of Figure 11, the main frequency components selected are 1×, 2×, 3×, 5×, 6×, and 8× SF and BF. Therefore, the parameter CNRs for the SES spectrum and the CNRc for the average CSES are obtained as follows:

(16) $$CNRs=\log\;(\sqrt{\sum_{j\in K_{s}}{\bar{\;CX\left(f_{j}\right)}}^{2}}/\sqrt{\sum_{i=1}^{Q_{s}}{\bar{\;CX\left(f_{i}\right)}}^{2}-\sum_{j\in K_{s}}{\bar{\;CX\left(f_{j}\right)}}^{2}}),\;\mathrm{with}\;K_{s}=\left\{1\times \;\mathrm{SF}\;\mathrm{and}\;1\times \;\mathrm{BF}\right\}\;,$$

(17) $$CNRc=\log\;(\sqrt{\sum_{j\in K_{c}}{\bar{\;CX\left(f_{j}\right)}}^{2}}/\sqrt{\sum_{i=1}^{Q_{c}}{\bar{\;CX\left(f_{i}\right)}}^{2}-\sum_{j\in K_{c}}{\bar{\;CX\left(f_{j}\right)}}^{2}\;}),$$

where $Q_{s}$ is the total number of cyclic frequencies in the selected SES band of Figure 8, $K_{s}$ is the ensemble of indices of cyclic frequencies in the selected SES band of Figure 8, $Q_{c}$ is the total number of cyclic frequencies in the selected average CSES band of Figure 11, and $K_{c}$ is the ensemble of the indices of cyclic frequencies in the selected average CSES band of Figure 11. As for the other CNRs in Figure 9 and Figure 10 and the CNRc in Figure 12 and Figure 13, the same routine can be carried out to update $K_{s}\;\mathrm{and}\;K_{c}$ in Equations (16) and (17), respectively.

In this paper, the measurements under 80%, 100%, and 110% flow rates at Sensor 15 were used for the CNR calculation and CFAR threshold selection. Then, new CNR and CFAR thresholds at other sensors could be adaptively regulated according to Equation (15). The higher the flow rate is, the higher the chance that cavitation occurs due to the characteristic curve of pump cavitation. We regulated the pressure of the vacuum pump to control the cavitation intensity of the testing pump. At one flow rate, the status is divided into 10 levels, named Status 1–10. Take the 110% flow rate, for example, where there is no cavitation at Status 1-7, incipient cavitation at Status 8, and severe cavitation at Status 9 and 10.

**Figure 15:**
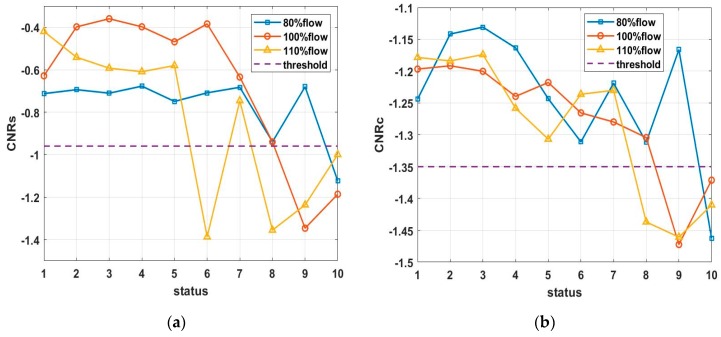

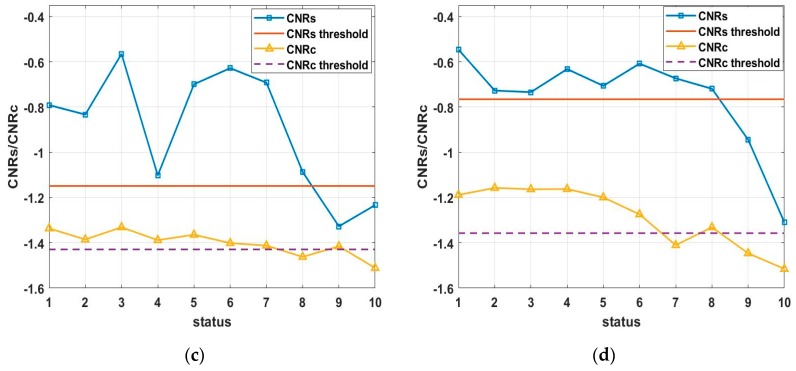
Feature parameters (CNRs and CNRc) against Constant False Alarm Rate (CFAR) thresholds at 80%, 100%, and 110% flow rates, with data obtained from Sensor 15, 17, and 19. False alarm rate remains at 5%. Detection rate of incipient cavitation is more than 90%. (**a**) CNRs curves and CFAR threshold along with 10 statuses at different flow rates, obtained from data at Sensor 15; (**b**) CNRc curves and CFAR threshold along with 10 statuses at different flow rates, obtained from data at Sensor 15; (**c**) CNRs curves, CNRc curves, and their CFAR thresholds, at a 100% flow rate, obtained from data at Sensor 17; (**d**) CNRs curves, CNRc curves, and their CFAR thresholds, at a 100% flow rate, obtained from data at Sensor 19. Note that the vertical labels denote the amplitude.

Based on the calculated CNRs and CNRc values in Equations (16) and (17), their CFAR thresholds were determined for experimental data, as shown in Figure 15a,b. Given a constant false alarm rate of incipient cavitation detection, $\;P_{fa}=5\%$, the CNR values were first matched with the normal status, incipient status, and cavitation status, respectively, by clustering analysis, and the CFAR thresholds $CNR_{0}$ among classified CNR values were trained by maximizing the classification distance, namely, by maximizing the detection rate of incipient cavitation. Finally, based on the trained CFAR thresholds at Sensor 15, the updated thresholds $CNR_{0}{}^{\prime }$ could be derived by Equation (15) to detect incipient cavitation measured at Sensor 17 and 19, as shown in Figure 15c,d.

The results in Figure 15a,b show that when cavitation develops into a severe status, there is a decreasing trend for both CNRs and CNRc curves. It can be noted that this trend is not strictly monotonically decreasing, because the cavitation-caused signals are not stationary during the pump operation, which causes some fluctuations of the CNR calculations in Equations (13) and (14). This also validates the proposed CAM model in Equation (1), for which, regardless of nonstationarity, cavitation-caused signals still have the cyclic amplitude spectrum; in this sense, the feature parameter CNR extracted from the SES or average CSES spectrum is capable of detecting incipient cavitation, as Figure 8, Figure 9, Figure 10, Figure 11, Figure 12 and Figure 13 demonstrate.

According to the classification of CNR values with the corresponding status, it is reasonable to make the CNRs threshold −1.00, i.e., when CNRs ≤ −1.00, its corresponding status is judged as cavitation. When the threshold of CNRc is −1.35, i.e., when CNRc ≤ −1.35, its corresponding status is judged as cavitation. Figure 14a shows that cavitation occurs at Status 10 at an 80% flow rate; at Status 9–10 at a 100% flow rate; and at Status 6, 8,9, and 10 at a 110% flow rate. In contrast, the detection result of Status 7 at a 110% flow rate is inconsistent with that of Figure 14b. As we pointed out, the CNR parameter was more precisely derived from the average CSES spectrum than the SES, so CNRc was more robust than CNRs. In Figure 14b, the CFAR threshold of parameter CNRc revealed that cavitation occurs at Status 10 at a 80% flow rate, Status 9–10 at a 100% flow rate; and Status 8, 9, and 10 at a 110% flow rate. There was no misjudgment at Status 7 at a 110% flow rate for the CNRc curve and its CFAR threshold.

The results in Figure 15c,d show that the CFAR thresholds were adjusted adaptively to deal with new data from Sensor 17 and 19 at a 100% flow rate. According to Equation (15), the selected CFAR threshold of CNRs for the data at Sensor 15 in Figure 15a can be used to infer the new CFAR threshold at Sensor 17, as shown in Figure 15c. Similarly, the selected CFAR threshold of CNRc at Sensor 15 in Figure 15b can help to derive the new CFAR threshold at Sensor 19, as shown in Figure 15d. CNRc is more confidently able to judge that Status 8 exhibits incipient cavitation, and severe cavitation probably happens at Status 10. These conclusions are in good agreement with the detection results obtained using Sensor 15 data in the same condition, as Figure 14a,b shows.

In addition, due to the different sensor positions, Sensor 17 tends to indicate that Status 7 could involve incipient cavitation, which produces an earlier waring than Status 7 at Sensor 15. According to Equation (13), if the false alarm rate is raised from 5% to 10%, the CFAR threshold of CNRc will be raised above the present line shown in Figure 15c. In that case, Status 7 would probably be detected as incipient cavitation. In this way, we can avoid any missing detection of incipient cavitation. The aforementioned conclusions also confirm the results of Figure 15d at Sensor 19 at a 100% flow rate. Due to the position of Sensor 19, Status 7 might be taken as incipient cavitation, which is more sensitive than Status 7 at Sensor 15 in the same condition, as Figure 11b shows.

Therefore, our proposed CNR feature parameters and CFAR thresholds can not only achieve more than a 90% detection rate of incipient cavitation and maintain a false alarm rate as low as 5%, but can also be used to adaptively regulate the CFAR thresholds in dealing with other sensor data, and finally make the optimal decision among different sensor measurements.

Additionally, it has been found that the result of Sensor 16 located at the inlet of the centrifugal pump is very different from that of other sensors. Neither the SES nor average CSES shows any features related to cavitation, so the calculated CNR values make wrong judgments, and the data of Sensor 16 can be regarded as invalid. In Figure 6, Sensors 12 and 13 located at the far end of the bearing are not suitable for judging the cavitation due to their close position to the motor and being located far away from the centrifugal pump. In contrast, the positions of Sensors 17, 18, and 19 are vertically, radically, and axially installed at the inlet flange, which is near to the pump body, so the data collected from Sensors 17, 18, and 19 are more reliable than those from Sensors 12, 13, and 16. Furthermore, the positions of Sensors 14 and 15 are directly mounted on the shell of the pump body, which is near to the inlet suction. That is why we selected Sensor 15 (or 14) as the reference, so that the proposed CNR and CFAR thresholds were firstly trained and selected by using cavitation data from Sensor 15, and then successfully applied to detect incipient cavitation measured at Sensors 17 and 19.

In sum, through various flow rates and different sensor data, the detection results of CNR parameters and CFAR thresholds are in good agreement with experimental conditions, so we can judge whether cavitation occurs by the proposed Autogram approach based on the CFAR detector.

## 6. Conclusions

Detecting cavitation occurrence during pump operation has significantly contributed to erosion prevention and predictive maintenance. However, most of the aforementioned state-of-the-art methods can hardly produce early warnings of incipient cavitation, and more importantly, can seldom guarantee a low rate of false alarm.

In this paper, an adaptive Autogram approach based on the Constant False Alarm Rate (CFAR) criterion has been proposed and validated for pump incipient cavitation detection. A cyclic amplitude model (CAM) has been presented to reveal the cyclostationarity and autocorrelation-periodicity of pump cavitation-caused signals. Then, the Autogram method has been improved for envelope demodulation and feature extraction by introducing the character to noise ratio (CNR) and CFAR threshold, which have been mathematically expressed by five steps in Section 3.1. To achieve a high detection rate, the improved Autogram introduces the CNR parameter to monitor cavitation-caused noises in the combined square-envelope spectrum (CSES) of pump vibrational acceleration signals. Moreover, to maintain a low false alarm, the proposed CNR parameter employs the CFAR detector to obtain adaptive thresholds in data processing from different sensor positions around one testing pump. By carrying out various experiments of a centrifugal water pump at 10 levels of cavitation intensities at different flow rates, the proposed approach is capable of extracting CSES features with respect to the CAM model, achieving more than a 90% detection rate of incipient cavitation, and maintaining a false alarm rate as low as 5%. The proposed approach is able to avoid missing detection and improve the detector robustness in pump cavitation diagnosis.

For the modeling of pump cavitation-caused signals, although it is time-varying, impulsive, and modulated by impeller rotation, the proposed CAM model is capable of reflecting the CSES characteristics of autocorrelation as follows: (1) CSES without cavitation: a family of comb components across the continuous spectrum, in which principal cyclic components (SF, BF, and their harmonics) sparsely distribute along low and middle frequencies, and a smooth carrier occupies high-frequency bands; (2) CSES with incipient cavitation: some high-order harmonics of SF and BF begin to be buried by cavitation-caused noise; (3) CSES with severe cavitation: noise clutters produced are so intensive that most of the harmonic components, even the BF, are buried. Additionally, there is no longer a family of comb components across the continuous spectrum. All these characteristics have been shown in Figure 1, Figure 3, and Figure 8, Figure 9, Figure 10, Figure 11, Figure 12 and Figure 13, and validated by real data of pump cavitation vibrations.

For the proposed CNR parameters to feature extraction, informative characteristic frequencies are selected through the combination of WT and PCA results, so that the ensemble K of the indice of characteristic frequencies can be determined from the SES or average CSES. Therefore, the proposed CNR parameters can be calculated adaptively by updating data from different sensors at various flow rates. All these features have been validated in Figure 8, Figure 9, Figure 10, Figure 11, Figure 12 and Figure 13.

For the adaptive CNR threshold to be involved in incipient cavitation detection, the CNR values are first obtained from the SES or average CSES spectrum by employing the Autogram method described in Section 3.1, and the CNR values with a normal status, incipient status, and cavitation status, respectively, are then matched by clustering analysis. Finally, the CNR thresholds are selected according to classified CNR values by maximizing the detection rate of cavitation, as the flowchart in Figure 4 shows. The detection results are consistent with the incipient cavitation characteristics modeled by the proposed CAM model, and have been validated by various experimental data, as shown in Figure 15. More importantly, the CFAR detector can regulate the threshold reasonably according to the different probability density functions p(z) of the noise in the SES or average CSES spectrum, as shown in Figure 5. Moreover, the CFAR threshold is able to be self-updated with different sensor data.

In sum, through various experiments of pump cavitation, our proposal has been confirmed to recognize incipient cavitation through a high detection rate and low false alarm. In future work, the NPSHi curve for pump cavitation diagnosis can be obtained, which is defined as the NPSH curve under incipient cavitation. In this sense, the pump operation following the NPSHi curve can avoid the cavitation problem from the very beginning stage, and greatly extend the pump life-span, as well as largely reduce the maintenance cost in the long run. To improve the robustness of data training, pump cavitation experiments should be carried out in more diverse conditions, so that the statistical distributions of cavitation-caused noise in the average CSES spectrum will be more reliable for a high detection rate. However, due to the danger of pump cavitation under extreme conditions, the proposed Autogram method based on CFAR detection should be improved by Bayesian inference techniques using small datasets.

## Figures and Tables

**Table 1:** Parameters of the centrifugal water pump used in this experiment.

Model	HZE80-50-250	Tag Number	A151-P-213A
Flow	52 m^3^/h	Head of delivery	84.65 m
NPSHr	2.1 m	Efficiency	61%
Speed	2950 rpm	Matching power	18.5 kW
Shell pressure	7.5 Mpa	Mass	120 kg
Bearing code	7308C7NU308	Machine seal type	C8B-55G7TD

Note: NPSHr means NPSH required.

## Nomenclature

ARMA	Auto Regressive and Moving Average
BF	Blade-passing Frequency
BP	Back Project
CAM	Cyclic Amplitude Modulation
CFAR	Constant False Alarm Rate
CMS	Cyclic Modulation Spectrum
CNR	Character to Noise Ratio
CNRc	CNR value obtained from CSES
CNRs	CNR value obtained from SES
CSES	Combined Square-envelope Spectrum
DFT	Discrete Fourier Transform
EMD	Empirical Mode Decomposition
FF	Fundamental Frequency
FK	Fast Kurtogram
HHT	Hilbert-Huang Transformation
IMF	Intrinsic Mode Function
MODWPT	Maximal Overlap Discrete Wavelet Packet Transform
NPSH	Net Positive Suction Head
NPSHa	NPSH available
NPSHi	NPSH incipient
NPSHr	NPSH required
PCA	Principal Component Analysis
RMSE	Root Mean Square Error
SE	Square-Envelope
SES	Square-Envelope Spectrum
SK	Spectral Kurtosis
SF	Shaft-rotating Frequency
SNR	Signal to Noise Ratio
SVM	Support Vector Machine
WPT	Wavelet Packet Transform
WT	Wavelet Transform
